# How does investment in research training affect the development of research networks and collaborations?

**DOI:** 10.1186/1478-4505-11-18

**Published:** 2013-05-20

**Authors:** Ligia Paina, Freddie Ssengooba, Douglas Waswa, James M M’Imunya, Sara Bennett

**Affiliations:** 1 Department of International Health, Johns Hopkins School of Public Health, 615 N. Wolfe St, Baltimore, MD 21205, USA; 2 Health Policy, Planning and Management Department, Makerere University School of Public Health, Mulago Hill Rd, Kampala, Uganda; 3 Independent consultant, P.O. Box 43994–00100, Nairobi, Kenya; 4 Institute of Tropical and Infectious Diseases, University of Nairobi, College of Health Sciences (KNH Campus), P.O. Box 19676–00202, Nairobi, Kenya

**Keywords:** Bibliometrics, Case study research, Collaborations, Global health, Kenya, Networks, Research capacity development, Research training, Uganda

## Abstract

**Background:**

Whether and how research training programs contribute to research network development is underexplored. The Fogarty International Center (FIC) has supported overseas research training programs for over two decades. FIC programs could provide an entry point in the development of research networks and collaborations. We examine whether FIC’s investment in research training contributed to the development of networks and collaborations in two countries with longstanding FIC investments – Uganda and Kenya – and the factors which facilitated this process.

**Methods:**

As part of two case studies at Uganda’s Makerere University and Kenya’s University of Nairobi, we conducted 53 semi-structured in-depth interviews and nine focus group discussions. To expand on our case study findings, we conducted a focused bibliometric analysis on two purposively selected topic areas to examine scientific productivity and used online network illustration tools to examine the resulting network structures.

**Results:**

FIC support made important contributions to network development. Respondents from both Uganda and Kenya confirmed that FIC programs consistently provided trainees with networking skills and exposure to research collaborations, primarily within the institutions implementing FIC programs. In both countries, networks struggled with inclusiveness, particularly in HIV/AIDS research. Ugandan respondents perceived their networks to be more cohesive than Kenyan respondents did. Network cohesiveness was positively correlated with the magnitude and longevity of FIC’s programs. Support from FIC grants to local and regional research network development and networking opportunities, such as conferences, was rare. Synergies between FIC programs and research grants helped to solidify and maintain research collaborations.

**Conclusions:**

Networks developed where FIC’s programs focused on a particular institution, there was a critical mass of trainees with similar interests, and investments for network development were available from early implementation. Networks were less likely to emerge where FIC efforts were thinly scattered across multiple institutions. The availability of complementary research grants created opportunities for researchers to collaborate in grant writing, research implementation, and publications. FIC experiences in Uganda and Kenya showcase the important role of research training programs in creating and sustaining research networks. FIC programs should consider including support to research networks more systematically in their capacity development agenda.

## Background and context

Research networks and collaborations are “loose social organizations” characterized by voluntary relationships, with varying degrees of formality, which change dynamically over time [1]. Networks allow for shared learning and comparative research, opportunities for coordination and implementing of international research projects, and widespread communication channels for the transfer of research to practice [1]. Given the complexity of contemporary global health topics, networks and collaborations across institutions and countries allow researchers to tap into specialist skills in different institutions and across national boundaries, to the benefit of those both in the South and in the North. Furthermore, research networks facilitate the recruitment of participants across multiple centers and countries and allow for the validation of findings across settings [2]. Networks and collaborations are also important in accessing research funds. Where domestic research funding is typically limited, as is often the case in low and middle income countries (LMIC) and particularly in Sub-Saharan Africa [3], research networks and collaborations, particularly between North and South institutions facilitate the development of competitive applications for international research funds. Research networks could contribute to building sustainable local and regional capacity, as well as to developing channels of information dissemination around topics on which the networks are focused [4].

Networks and collaborations are increasingly recognized as important for harnessing multiple, different disciplinary perspectives to tackle today’s complex issues in health research such as attainment of the Millennium Development Goals [2]. The promotion of research networks and partnerships in global health is one of the nine key requirements outlined by the African-led Initiative to Strengthen Health Research Capacity in Africa [5]. The significance of research and network collaborations was also underlined by the 2008 Global Ministerial Forum on Research for Health’s Call to Action, which urged national governments “to develop mechanisms and tools to enable effective inter-sectoral, inter-ministerial, and inter-country research collaboration and coordination to address complex health challenges,” as well as “to work through regional alliances to advocate for research, establish networks of researchers and regional centres of excellence, ensure coherent and sustainable funding, improve education and career opportunities in research and research management, and strengthen harmonization of regulation and ethical conduct” [6].

National or regional research networks often develop around a common research area for purposes of collaboration and sharing information [7,8]. Research training programs can either have networking as a deliberate element to stimulate mentorship and research collaboration opportunities [9], or use a network structure to provide members with training opportunities, both short-term (such as workshops and certificate courses) and long-term (such as Masters, PhD, or other degree earning courses). Researcher training is key to developing the capacity of individual researchers, but the extent to which these training programs contribute to broader aspects of research capacity such as network-level effects, is underexplored. It is possible that in contexts where research capacity is underdeveloped, as is the case for many LMIC institutions, research training programs could provide an entry point for the development of such research networks [10]. We contribute to current insights on research networks by examining whether investment in research training for LMIC researchers through the Fogarty International Center’s (FIC) training programs influenced the development of research networks and collaborations, and, if so, which programmatic and contextual factors were most critical in this endeavor.

The FIC at the National Institutes for Health (NIH) is dedicated to supporting and facilitating global health research conducted by US and international investigators, building partnerships between health research organizations in the US and abroad, and training the next generation of scientists to address global health needs. FIC supports biomedical research and training at LMIC organizations through grants that are either awarded to US universities which partner with LMIC ones or, increasingly, directly to LMIC organizations. FIC currently administers 23 programs, 17 of which are research training programs. Scholarships represent the main component of these training programs. Training ranges in duration and scope, from short-term certificate programs aimed at providing participants with focused skills, to long-term training such as Masters and Doctoral programs. FIC degrees were typically for clinical and biomedical research, often related to HIV/AIDS, but also to other topics, such as bioethics, operational and health services research, and infectious diseases. The training programs also had a strong mentorship component, linking students from LMIC organizations with US and local mentors. For example, the Fogarty International Clinical Research Scholars program (FICRS) paired up students from US and LMIC organizations to partner on research projects. Although FIC requests for applications provide general guidance on the vision of the training programs, the US-based universities in conjunction with the LMIC partner organizations design the training program, resulting in varied emphasis on training components, such as long-term and short-term training, among the various programs. The oldest FIC program, the AIDS International Training and Research Program (AITRP) dates back to 1988 and, by 2004, had trained a total of approximately 1,400 researchers [11]. Although more recent estimates are not available at this time, the AITRP program continued to train more researchers until the recent re-organization of FIC research training programs.

In this paper, we focus specifically on research training programs at Makerere University in Uganda and at the University of Nairobi in Kenya, which have both benefitted from long-standing support from FIC. We explore whether and how FIC’s investment in research training contributed to research network development and we seek to identify facilitating factors. Because FIC support and the emphasis on different components varied among programs, we cannot draw conclusions about causal mechanisms through which program components influenced research networks, or compare the contribution of different types of interventions, individually or in combination.

Table 1 lists the main FIC research training grants awarded in Kenya and Uganda. While multiple grants are listed, the most significant in terms of resources invested in the two countries are the AITRP awards to Case Western University and Johns Hopkins University for work in Uganda, and the University of Washington grant for work in Kenya. According to FIC records we estimate that by early 2010, 135 individuals had completed long-term (6 months or more) training under FIC programs in Uganda, and 82 individuals had completed similar training in Kenya. Much of this training had taken place under AITRP: almost 60% of those trained in Uganda had been under the AITRP program and almost 50% in Kenya.

**Table 1:** Summary of FIC research training programs at case study universities, as of 2011

**US partner**	**Fogarty international center program**	**Degrees offered /nature of collaboration**	**Timeline**
**University of Nairobi, Kenya**			
University of Washington	AIDS international training and research program	Masters, PhD, shortterm, non-degree, post-doctoral training	1988 - present
Case Western Reserve University	AIDS international training and research program	Masters, PhD, long-term post-doctoral, short-term post-doctoral, non-degree, summer courses, in-country advanced research projects and workshops	1988 - present
US Walter Reed Army Institute of Research	ABC malaria	Fellowship program for four physicians	2003 - 2004
Harvard	AIDS international training and research program	Long and short-term training	1988 - present
University of Cape Town	Bioethics	Networking and short-term training	2003 - 2010
UCSF	Global infectious disease	Certificate programs	2006- present
Vanderbilt University	Fogarty international clinical research scholars	Pairing with US-based scholar, short-term training in the US, mentoring	2007-present
**Makerere University, Uganda**			
Case Western Reserve University	AIDS international training and research program	Masters, PhD, short-term, non-degree, post-doctoral training	1988 - present
	Bioethics (multi-country)	Masters	2000 - 2006
	AIDS international training and research program	Masters, PhD (sandwich), US and Uganda-based non-degree training	2002 - present
Johns Hopkins University	AIDS international training and research program	Masters, PhD, long-term post-doctoral, short-term post-doctoral, non-degree, summer courses, in-country advanced research projects and workshops (Uganda + region)	1988 - present
	Population health (POP)	At Johns Hopkins University: Masters, PhD, post-doctoral; At Makerere University School of Public Health: Masters, PhD, non-degree (Uganda + region); field training at Rakai and curriculum development	2000 - present
	Bioethics (Multi-country)	1 year program, 1 month training	2000 - present
University of California Berkley/University of California San Francisco	AIDS international training and research program	Masters, short term non-degree training	1988 - present (in Uganda since 2004)
	UCSF/UCB international malaria research training program	Trainees linked to research project given opportunity for Masters	2000 - present
	Makerere University - UCSF international malaria clinical, operational and health services research training programs	At Makerere: Masters	2006 - present
		At partner institutions: UCB Masters, London School of Tropical Medicine and Hygiene Masters and PhD	
		Short-term non-degree specialized courses	
Baylor University	AIDS international training and research program	Long and short-term training, building center of excellence	2002 - present
University of Washington	AIDS international training and research program	Long and short-term training	1988 - present (in Uganda since 2007)

## Methods

In 2009, FIC commissioned a research team at Johns Hopkins University’s School of Public Health in Baltimore, MD, to carry out two country studies in Sub-Saharan Africa, focused on two institutions which received substantial, long-term support through FIC research training programs since the late 1980’s: Makerere University’s College of Health Sciences in Uganda and The University of Nairobi’s College of Health Sciences in Kenya (UoN). The largest active FIC program in these institutions was AITRP, however, we also examined other, smaller programs that provided long-term research training (*e.g.*, FICRS, Bioethics). The overall purpose of the case studies was to assess the long-term effects of FIC research training programs on research capacity. The network level represented one of the levels at which FIC’s impact was assessed.

For this qualitative research project, the team employed a case study approach, with the local universities serving as the unit of analysis. Ethical approval for this study was obtained from the Institutional Review Boards at Johns Hopkins University School of Public Health, the Makerere University School of Public Health, and the University of Nairobi. Table 2 summarizes the qualitative data collection procedures from the first part of this study. Semi-structured interviews and focus group discussions with FIC alumni within and outside of the local universities, as well as with university leadership and policy-makers, were conducted in Uganda during April-May 2011 and in Kenya during September 2011 through a joint partnership between the Hopkins research team and our local collaborators. In Uganda, all data collection was conducted in person as most of our respondents were based in or were able to conveniently be in Kampala during our data collection. In Kenya, we conducted phone interviews with respondents who were not in Nairobi. Our respondents were sampled from all individuals for whom data was available in FIC’s internal systems, who had completed long-term training (*i.e.*, training longer than 6 months, usually Masters or PhD) before the data collection period. Because our case studies were exploratory, they were not designed to capture systematic differences between FIC programs’ design and implementation. Nevertheless, we tried to include respondents from all the various FIC programs present in our settings. All respondents provided written consent prior to their participation in our study.

**Table 2:** Summary of data collection in Uganda and Kenya

**Form of interview/respondent**	**Uganda interviewees**	**Kenya interviewees**
Focus group discussions (FGDs)	6 (participants = 19)	3 (participants = 10)
**TOTAL FGD participants**	**9 FGDs (29 participants)**	
Interviews with principal investigators	5 (US and Ugandan)	3 (US and Kenya)
Interviews with FIC trainees	6 (4 university-based and 2 non-university-based)	20 (7 based at UoN and 13 outside UoN)
Interviews with Institutional leaders	5	5
Interviews with policy makers	4	5
**Total in-depth interview participants**	**53 participants**	

Our interview and focus group discussion guides covered a number of topics related to the long-term effects of FIC investment in research capacity, including specific questions concerning how FIC training contributed to the development and maintenance of research networks. All interviews were recorded. In Uganda, due to budgetary constraints, only those recordings for which detailed notes did not exist were transcribed. In Kenya, all of the recordings were transcribed. We used thematic analysis methods to analyze the qualitative data. Thematic analysis involves becoming familiar with the data collected through interviews and focus group discussions, developing a coding structure which relates the data to the research question, and the identification and discussion of themes or patterns which emerge from the data. The Uganda analysis was conducted manually to facilitate the participation of local collaborators in coding of the text. All Kenyan recordings were transcribed and the analysis was assisted by the Atlas.ti 6.0 program. Similar coding schemes were used for both of the case studies.

In the second phase of this study, we complemented our qualitative findings on networks with a bibliometric analysis focused on the prevention of mother-to-child transmission of HIV/AIDS (PMTCT) and male circumcision (MC). The purpose of the bibliometric analysis was to confirm whether the networks which our respondents described could also be identified in the scientific activity stemming from the two universities. The two research topics were perceived by our respondentsas having strong networks and collaborations formed around them with the help of FIC research training program. Both of these research topics have been supported by long-term AITRP grants. While not comprehensive, this focused analysis provided a flavor of the nature of research collaborations on these two topics.

The bibliometric analysis was based on searches for research articles constructed in PubMed and run in September 2011 for Uganda and April 2012 for Kenya, and were initially inclusive of all articles until the present. We manually excluded some publications, such as letters to the editor, as they did not capture research collaborations. We extracted data on the number of publications for all Ugandan and Kenyan authors for the available years, noting their institutional affiliation, whether they were first authors, and/or former FIC trainees. We developed basic tallies of these numbers in Excel.

In the final step of our analysis, we used the same PubMed searches to illustrate the resulting networks with the tools available on GoPubMed (gopubmed.org). GoPubMed is a free online service that can generate network visualizations based on PubMed search results. Using PubMed citations, it extracts authors’ names and creates a map illustrating connections based on frequency of collaboration or co-authorship. The frequency of collaboration is displayed using connecting lines of different thickness for either 1, 2, 3–5, or more than 6 collaborations. For each of the figures we obtained from GoPubMed, we identified and highlighted actors who were relevant to our case studies, *i.e.*, FIC trainees, Ugandan or Kenyan principal investigators, and US principal investigators.

## Results

### Did FIC contribute to network development?

A significant number of trainees in both Uganda and Kenya remained in their country of origin after completion of FIC training. In Uganda 135 individuals had received long-term training, of which we could trace the whereabouts of 126; 113 of these individuals were still in Uganda, and 66 of them worked for Makerere University. In Kenya, 82 individuals had received long-term training, of which we could trace 72. Sixty of these individuals were still in Kenya and 41 of them were employed by the University of Nairobi, the Kenya Medical Research Institute, or the Kenyatta National Hospital. Across the institutions studied in Uganda and Kenya we identified research networks for sharing information, for coordinating and implementing research, and for informing policy. The latter type of research collaboration is discussed in greater detail elsewhere [12] and therefore is not the focus of our discussion here. Research networks for sharing information and implementing research centered on relationships between US-based FIC institutions and African institutions, involving both current and former trainees. Such research networks were initiated during the training program, but generally were maintained after the training was completed. Almost all participants from both countries explained that they maintained positive working relationships with their US-based FIC collaborators even after the conclusion of the training program. This included both regular communication regarding professional development, as well as collaborations on proposal writing and grant implementation. Such research collaborations were strongest for the large AITRP programs, *i.e.*, Case Western University and Johns Hopkins University in Uganda, and the University of Washington in Kenya, which was closely associated with the University of Nairobi’s Department of Pediatrics and Obstetrics and Gynecology. According to our respondents, networks among FIC trainees were most evident in relation to the PMTCT research carried out under the University of Washington grants. Former trainees in the University of California – San Francisco’s (UCSF) AITRP program also cited on-going, long-term relationship with their US counterparts.

“Contacts that I made then like Dr. [name of UCSF collaborator] we have been working together for the last 17 years. It was a very good partnership that has continued to grow and also the networks that I developed then even from the University of Washington I have continued to collaborate with others like [names of US collaborators].” Trainee outside UoN, Kenya

In both Uganda and Kenya, trainees from smaller FIC programs such as FICRS program and the Fogarty Bioethics program, which selected a handful of individuals from multiple countries to participate in research training programs, had less of a relationship with US-based collaborators, both during and after the training period. These weaker links were most probably due to the nature of these programs, which were shorter in length and included only short periods of interaction between trainees and their US mentors. During training, some networking with FIC trainees from other countries or US-based institutions was present. However, according to respondents from both FICRS and the bioethics program, a strong research network and sustained interactions, after the training period concluded, were not achieved.

In-country research networks were also discussed by our respondents, however research relationships between FIC trainees and other local researchers, differed by country. In Uganda, FIC alumni and other Ugandan researchers were brought together through the Uganda Society for Health Scientists (USHS)^a^. The USHS is a formal research network that was established in 1999 through the initiative of the FIC principal investigator from Case Western, together with a number of Fogarty alumni now in senior positions at Makerere University. The USHS is open to all researchers in Kampala and currently counts around 500 members. It also houses the Uganda Fogarty Alumni Association, a subgroup of about 60–70 former trainees that has been established within USHS since 2006. The USHS has played an important role in several respects related to network development. First, it provided former trainees with a formal platform for collaboration and continued capacity development, a sense of belonging in the post-training period, as well as the opportunity to pass on their skills to others. Second, through research training opportunities – many led by former FIC trainees – periodic journal clubs on key research topics, and annual conferences, the USHS fostered the development of an in-country networking of researchers and most importantly, contributed to the amplification of the research training through sharing of knowledge from FIC.

A similar organization or arrangement was not present in Kenya. Outside of the University of Washington AITRP, and to some extent the UCSF one, Kenyan FIC trainees were not always linked up with their FIC peers or other international researchers. Respondents felt that they had often made strong bonds with trainees from other countries during their training period, yet there had been no structures to help them maintain these linkages upon returning to Kenya.

*“When we were students it [our network] was so powerful really, after we parted everyone got scattered.”* University trainee, Kenya

### What mechanisms and approaches contributed to network development?

Based on the data we collected, it is evident that there are several mechanisms and approaches through which FIC contributed directly to fostering research networks. Most of these approaches to fostering research networks were relatively informal, although the USHS provides an example of a formal network to have emerged from FIC support.

In Uganda, many former FIC trainees described how the program had provided them with networking skills and opportunities which, in turn, helped them to extend their networks. Through engagement with US-based collaborators and faculty – which often represented their first exposure to research collaborations – FIC trainees learned how to network during the period of their training. One of the institutional leaders said that:

*“It [FIC training] can open out how other people work, so they learn how to network with people from other countries…..So you find that people who have been through the program are more likely to grab onto a visiting scientist and link with them.”* Institutional leader, Uganda

A University-based FIC alumnus explains how these networks were then developed beyond the training programs.

*“Yah, well both at the individual and organizational level I have improved tremendously my networking. I now network with colleagues in Europe, but also colleagues within Africa but mainly through research and capacity building*” University trainee, Uganda

Another factor which contributed to the development of networks beyond the duration of the actual training programs is FIC’s long-standing reputation in research training. The fact that FIC was renowned to provide high quality research training inspired trust among foreign collaborators that FIC trainees’ had the necessary scientific skills to produce rigorous research. Accordingly, FIC trainees were often sought out for collaboration by investigators from elsewhere.

*“Those people have become a target as it were, for investigators elsewhere. They look to them for, you know, as co-investigators or whatever other role. But they are sought after to participate in research.”* Institutional leader, Uganda

Last, but not least, NIH research funding was a key driver both for the formation and long-term sustainability of research networks in both countries. Very little local research funding was available in Uganda and Kenya. Therefore, networks with US-based and other international institutions were a key entry point for accessing research funds. FIC alumni, as they transitioned from the training programs to their local research careers, applied for NIH research grants, usually in collaboration with colleagues based at US institutions, whom they had met through FIC training and related research activities. Furthermore, FIC's long-term investments in research training programs facilitated the establishment of on-going research teams working on NIH funded research projects in both Uganda and Kenya. The existence of local research teams facilitated the return of FIC trainees to their home institution, where they could find employment within these on-going projects. Therefore, NIH research grants reinforced the networks that were developed through the FIC training programs.

### Effects of research networks in Uganda and Kenya

Respondents cited a number of different ways in which the research networks developed through FIC had benefitted them. One of the main advantages of research networks, as alluded to above, was that such networks allowed former trainees to bid collaboratively on requests for proposals issued by NIH and other research funders. Respondents described how their networks had helped to enrich their thinking and open their minds to different perspectives. At Case Western University, FIC trainees had been supported to invite speakers to the school, and one described the great richness of different perspectives and ideas that this offered.

*“On top of having the knowledge, skills, and the diploma – networking plays a very big role. I look on the first three things as being the engine, wings, and fuselage of the plane – but networking is the fuel that helps you reach a higher altitude.”* Makerere University trainee

In Kenya, support from existing research networks, including researchers at other universities was seen as being particularly beneficial during the period directly after return from training. Respondents identified this period as a particularly difficult time, especially for those not lucky enough to receive strong support for job placement. Under these circumstances, connections to external colleagues helped trainees to have the courage to pursue their own independent lines of research.

Not all respondents shared the same positive views about networks. Generally, research networks – both during and beyond training – were perceived to be more open in Uganda than in Kenya, although within both countries, HIV/AIDS-related research networks were viewed to be relatively closed. Respondents from Uganda who had been through the FIC program or who had been closely associated with it, very much appreciated the benefits of networking, but interviews with some institutional leaders showed that the FIC network was sometimes viewed as a closed “club”.

*“If you went around the College today and you asked each of the faculty members “What do you know about Fogarty International?” my guess is you would probably get 10 out of 100 telling you “Yes, I know something about Fogarty”, simply because, I think the networking is closed.”* Institutional leader, Uganda

Similar to Uganda’s situation, respondents in Kenya thought that the FIC research network was closed - non-UoN trainees explained that while senior researchers have a strong network, junior researchers felt that those networks were not available to them. UoN’s network was strongly focused around the programs associated with the Department of Obstetrics and Gynecology, leading to some frustration among outsiders to this group.

*“The people who are here, for example the University of Washington family, know one another. [B]ut is there a way that other Fogarty scholars or other people who have benefited from Fogarty training can network outside the institutions where they received the training?”* Former trainee outside of UoN, Kenya

In contrast with Uganda’s networks, Kenya’s networks outside of UoN, and especially outside of HIV/AIDS work, were also very diffuse. Individual FIC trainees, even within the same institution, seemed to be a part of different research networks, both international and regional. The absence of strong networks within the research institutions mentioned by our respondents in Kenya hints at the fact that former FIC trainees may not have access to multidisciplinary research and other benefits which stem from research networks based at one’s institution. The diffuse networks might be explained by the fact that Kenya houses a large number of organizations where former FIC trainees are based, but only few of these organizations have a critical mass of trainees. Additionally, the diffuse networks our respondents described may also be related to a reliance on informal relationships driven by individuals, rather than institutional linkages.

### HIV/AIDS research networks: a closer look at PMTCT and MC networks

The focused bibliometric analysis was conducted to assess whether respondents’ perceptions of the nature of the networks which emerged from FIC training programs, were supported by patterns of publication. The final set for analysis included 145 publications for PMTCT and 50 for MC in Uganda, and 83 publications for PMTCT in Kenya and 59 for MC (Table 3). The number of all publications produced on PMTCT and MC increased significantly between the late 1980’s and early 1990’s to 2011, demonstrating that these areas were indeed significant. For example, in the 1990’s there were either 1 or 2 papers published per year on PMTCT and MC in Uganda. By the 2000s, up to 19 PMTCT articles and 14 MC ones were published per year in Uganda. In Kenya, PMTCT and MC research was published mostly since 2000, but similar trends appear. Whereas there were only 1 or 2 articles published per year on these topics during the early 2000s, by the end of that decade, PMTCT publications rose to about 17 per year, and MC publications ranged between 6 and 13 per year. Local authors contributed to most of the articles examined. FIC trainees also significantly contributed to the articles we examined. In Uganda, at least one FIC trainee contributed to 27% of the PMTCT articles and 40% of the MC articles we found. In Kenya, at least one FIC trainee contributed to 41% of the PMTCT articles and 12% of the MC articles we examined.

**Table 3:** Summary of bibliometric analysis data

	**Uganda**		**Kenya**	
**Publication characteristics**	**PMTCT**	**MC**	**PMTCT**	**MC**
Total number of articles	145	50	83	59
Articles with any contribution from a FIC trainee	39 (27%)	20 (40%)	34 (41%)	7 (12%)
Articles first-authored by local authors	28 (19%)	11 (22%)	24 (29%)	1 (2%)
Articles first-authored by FIC trainee	9 (6%)	9 (22%)	6 (7%)	0 (0%)

First-authorship among local authors, however, remained very low in both countries for the entire period examined. In Uganda, 28 (19%) of all the PMTCT articles and 11 (22%) of the MC ones were first-authored by local authors. In Kenya, 24 (29%) of the PMTCT and 1 (2%) of the MC articles had local first authors. The low frequency of first-authorship throughout the period hints at potential underlying asymmetries in research leadership between foreign and local researchers within the research networks. Because the total number of articles on these focused topics was low, we could not draw any final conclusions about authorship trends comparing FIC trainees with non-trainees.

Based on institutional affiliations, Makerere University researchers dominated the Ugandan publications on PMTCT and MC. Makerere University is Uganda’s main hub for research and also the focal point of most FIC programs in Uganda, and therefore many FIC alumni currently work there. Within this hub, programs such as the Rakai Health Sciences Program or the Makerere University-Johns Hopkins University (MU-JHU) collaboration exhibited tighter research networks. In Kenya, the University of Nairobi played a much less dominant role, with authors being affiliated with several other Kenyan research institutions, such as the Kenya Medical Research Institute. This finding confirms our respondents’ perspectives that networks in Uganda were more cohesive and focused around Makerere, whereas networks in Kenya were more diffuse.

**Figure 1:**
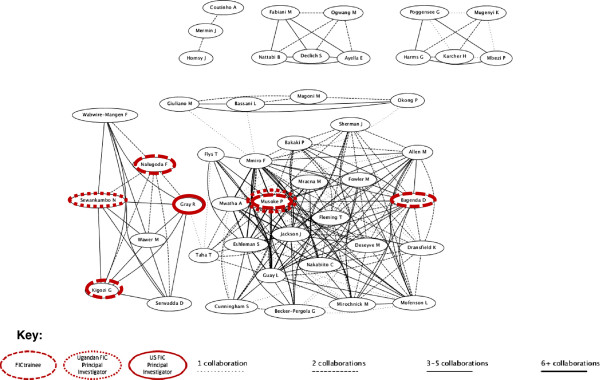
PMTCT Uganda research network.

**Figure 2:**
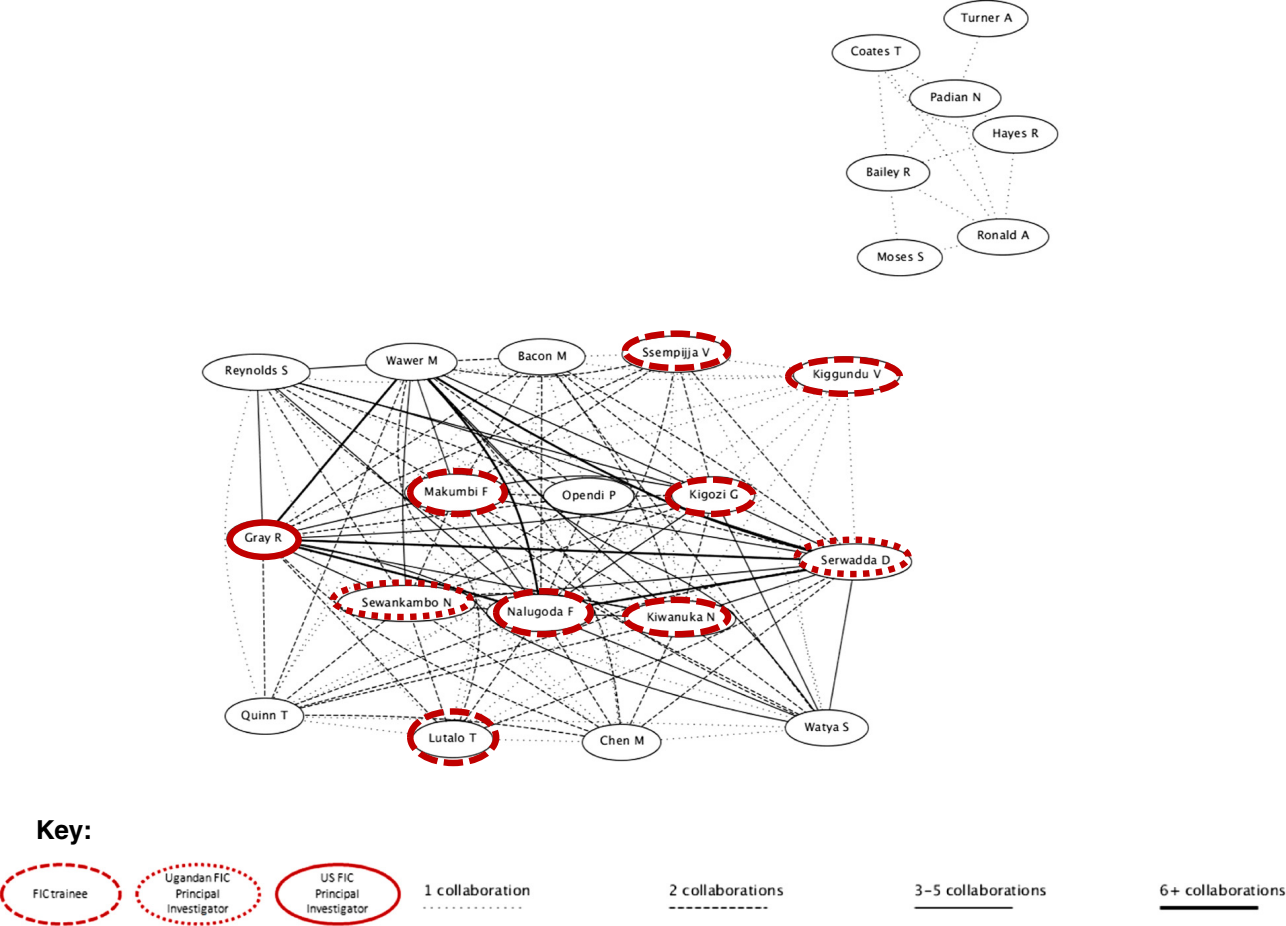
MC Uganda research network.

**Figure 3:**
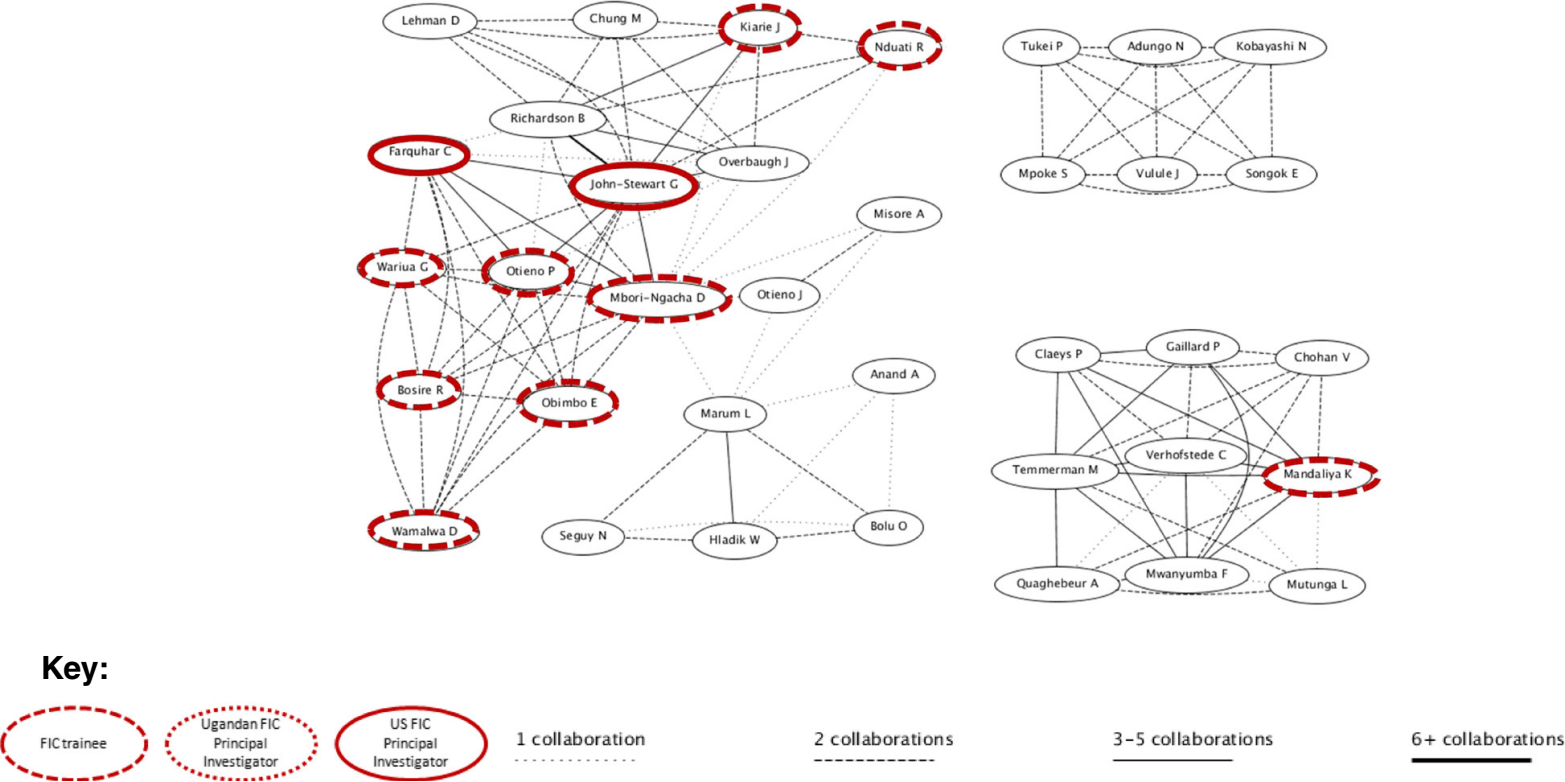
PMTCT Kenya research network.

**Figure 4:**
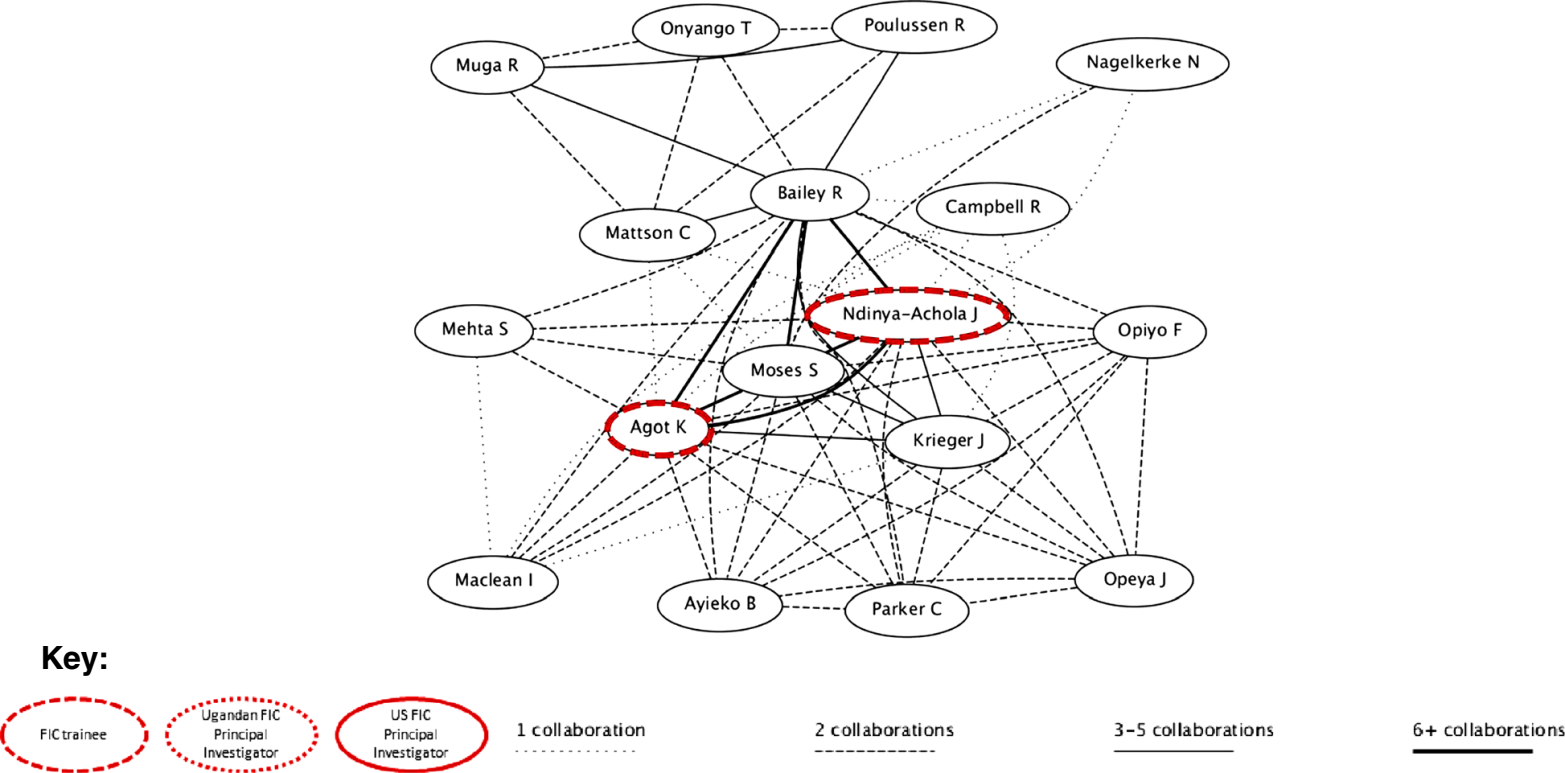
MC Kenya research network.

Our network visualizations from http://www.gopubmed.org present a similar picture (Figures 1, 2, 3, 4). These figures illustrate the clusters of authors who collaborated on PMTCT and MC articles, respectively. The research networks for both PMTCT and MC in Uganda appear to be more dense and connected compared to those in Kenya (the strength of the lines depicts the number of articles co-authored by any particular pair of authors and the FIC trainees and principal investigators are marked). In Uganda, the PMTCT network centers around the collaborations between Johns Hopkins University’s Eshelman and Guay and Makerere University’s Musoke and Mmiro^b^. For MC, the network is much more cohesive, likely because most research on male circumcision was developed at the Rakai Health Sciences Program^c^. FIC trainees were especially involved in the MC network.

In Kenya the FIC network was concentrated around the PMTCT work led by the University of Washington’s AITRP program (Figure 3). The major cluster in the PMTCT research network is around papers by Farquhar and John-Stewart, the two US-based principal investigators of the University of Washington AITRP program. This cluster displays a dense network of FIC trainees and, typically, multiple collaborations. The members of this network are a mix of trainees from UoN and other research institutes in Nairobi. No local principal investigators were involved in these PMTCT publications. The same type of network was not identified in the area of MC research. As displayed in Figure 4, Kenya was less of a hub of MC research than Uganda, and had only a couple of FIC trainees involved.

## Discussion

The two case studies identified several contributions by FIC research training programs to research network development. FIC research training program primarily contributed to the development of networks between individuals based in LMIC universities and those based in the US partner institutions. These were initiated during the duration of the training period, and were often maintained afterwards. While the kernel of the network typically centered on the partnership with the US university that held the research training grant, over time additional new linkages were formed. Former FIC trainees who were active in the research networks placed great value on such networks, and recognized the critical role they had played in their career development and ability to access research funds.

FIC’s contribution to the development of broader networks, such as those within and between local institutions in the same country, or among researchers or institutions based in two or more LMIC countries, was limited, particularly in Kenya. The three main factors influencing the degree to which FIC’s programs contributed to network development were the nature of support to network development, the organizational and research culture of LMIC entities, and the structure and longevity of FIC engagement. We summarize key points concerning each of these factors in turn.

In the absence of formal, sustained support to research network development, informal research networks among individuals develop *ad hoc*, based on individual champions. While the initial networks between US and LMIC institutions were developed as formal collaborations through the research training programs, they were sustained informally upon the completion of FIC trainings. These informal relationships were typically nurtured through the initiative of US-based principal investigators and highly motivated FIC alumni, who often ended up in senior leadership positions at their home institutions. Informal collaborations in both Uganda and Kenya supported the writing of publications and grant proposals, as well as research implementation. A notable exception is the USHS, a funded, formal research network which acts as a forum for researchers across Uganda, as well as for FIC alumni.

The development of research networks in Kenya and Uganda mirrored the research context and organizational cultures in which FIC intervened. Traditionally, health research in Uganda has been focused around Makerere University, while in Kenya there are multiple universities and research institutions that are scattered around the country. Additionally, Makerere University’s research culture is stronger than at the University of Nairobi, due in part to the critical mass of researchers to which FIC contributed, but also to the long history and strong research focus of Makerere as well as outstanding research leadership. At the University of Nairobi, the value of health research has received more recent recognition (see, for example, the speech of the Vice-Chancellor to the graduating class in 2011 [13]). Accordingly the networks that developed at Makerere were stronger, broader and more densely connected, compared to those that developed at the University of Nairobi.

This symbiotic relationship between research networks and organizational culture suggests that investment is needed in both network development and organizational development. Further, network development approaches should be tailored to specific organizational characteristics. For example where research culture is relatively nascent it may make more sense to focus on developing smaller more closed collaborations, so long as these collaborations are encouraged to expand and diversify over time.

Sustained engagement through training programs and the synergy with research funding opportunities can provide a powerful combination for research network development, but not without potential pitfalls. Training programs such as AITRP and ICOHRTA, both within UoN and Makerere, have now continued for nearly 25 years and in the three main AITRP programs studied there has also been continuity in terms of the US principal investigators involved. Additionally, in both Uganda and Kenya, NIH research grants acted to build and reinforce the networks which were developed through the FIC training programs. While this was generally a very positive development, it meant that already strong areas were further reinforced by external grant funding and little was done to develop research networks in more “neglected” areas. Also, it meant that networks in certain research areas, in which NIH funds were received and implemented by the same group of individuals (*e.g.*, HIV), came to be seen as “closed clubs.” In both Uganda and Kenya, some individuals outside of FIC networks felt somewhat excluded from these networks.

A persistent challenge in network development relates to differing levels of power between US and LMIC researchers in the networks. The dynamics within North–south research collaborations usually reveal structural inequalities, by which Northern researchers hold more influence on research agendas, leadership, successful funding applications, and publications [14]. An example of this is how the number of publications first authored by African researchers in the two areas we examined for the bibliometric analysis remained low throughout the period of analysis despite significant contributions of African researchers to the publications. Although not explored in our case studies, the research networks developed also appear to have provided significant benefits to collaborators in the north, by enhancing their ability to access skilled southern researchers who contributed to the quality of studies done in several respects. As research networks evolve they need mechanisms to address such power imbalances, and research funders potentially have a significant role to play in assisting with this process.

Although we cannot extrapolate the findings from the two case studies to other countries, the mechanisms through which FIC’s investments in research capacity contributed to research network development, as well as the challenges and issues which arose along the way resonate with those found in other low and middle-income settings. Our findings confirm that certain elements identified in previous studies as key to network development, such as providing specific funding to the development of networks and the establishment of coherent themes for collaboration [4,10,15], worked as facilitators for network development in FIC’s research training programs. For example, funding to support USHS appears highly successful, and networks were strongest when focused on specific research topics, such as HIV/AIDS.

### Limitations

A potential bias in our study arises from the fact that our respondents included mostly individuals who have benefitted from FIC training and associated NIH research funding. Although there is potential that these respondents would feel generally positively about FIC, we found that many of them were candid about the weaknesses and challenges associated with the FIC training programs either in their experience or that of their peers. Our case studies were limited by the fact that primary data collection for a social network analysis, which was part of our initial study design, was dropped soon after data collection began in Uganda due to the fact that survey implementation took much longer than anticipated and resulted in considerable burden on our respondents. Because of these logistical challenges, the social network analysis component was removed from the Kenya case study as well. In the absence of the network survey, we were not able to conduct a detailed social network analysis or to produce quantitative network measures such as density and centrality, and relied solely on the additional analyses described in the earlier sections of this publication.

## Conclusions

We observed the development of local research networks when funding from research training programs, such as FIC’s, was available to sustain networking activities. A critical mass of trainees working in a particular research area or at a particular institution helps to grow these networks, as observed with the USHS in Uganda.

FIC and other supporters of research trainings should be mindful that research networks are less likely to evolve when research training efforts do not place early and sustained emphasis on creating and maintaining linkages among trainees, particularly when the trainees themselves are thinly scattered across multiple institutions and sustained mechanisms to bring them together are lacking. Along the same lines, too much focus on developing networks in a particular research area, can at times be to the detriment of other areas, and has implications for the long-term inclusiveness and sustainability of networks in general. The case study in Kenya illustrates this challenge around its HIV/AIDS research network. Implementing research training programs in settings where research grants were available was key to sustaining research collaborations and promoting the emergence of new networks.

The future research agenda on networks should focus on tracking network development over time. Social network analyses would provide a sophisticated view of networks in a country and permit the creation of metrics by which research networks across settings could be compared. Funders of research training programs who are interesting in supporting network development should consider explicitly including such an objective in their program design, supported by adequate metrics to track progress over time.

## Endnotes

^a^See the Uganda Society for Health Scientists website for further information (http://www.ugshs.org/)

^b^The smaller collaborations surrounding this one represent either work funded by other donors (*e.g.*, Italian donors for Bassani et al., or GTZ for Harms et al.) or expansions in the JHU/Makerere collaboration (*e.g.*, through research on PMTCT at the Rakai Health Sciences Program through Gray, Serwadda, et al.)

^c^The smaller network in the top right-hand corner of the figure represents research conducted through an international collaboration for reviews in which data from Uganda was mentioned, but Makerere University or Ugandan researchers were not involved.

## Abbreviations

AITRP: AIDS International training and research program; FIC: Fogarty International Center; FICRS: Fogarty International clinical research scholars program; LMIC: Low and middle income countries; MC: Male circumcision; MU-JHU: Makerere University-Johns Hopkins University collaboration; NIH: National Institutes for Health; PMTCT: Prevention of mother-to-child HIV/AIDS transmission; UCSF: University of California – San Francisco; USHS: Uganda Society for Health Scientists; UoN: University of Nairobi

## Competing interests

The authors declare that they have no competing interests.

## Authors’ contributions

SB is the principal investigator in the overall study and proposed the scope for this article. LP and SB were engaged in conceptualizing and preparing the first draft of this paper, incorporating other authors’ comments, and preparing for publication. FS, DW, and JM were engaged in editing and providing comments on the draft. All authors were involved in the data collection for the case studies. All authors read and approved the final manuscript.

## References

[B1] United Nations Conference on Trade and Development Making North–South Research Networks Work 1999 Geneva: United Nations

[B2] Dockrell HM Presidential address: the role of research networks in tackling major challenges in international health International Health 2010 2 181 185 10.1016/j.inhe.2010.07.004 24037698

[B3] Kilama WL The 10/90 gap in sub-Saharan Africa: resolving inequities in health research Acta Trop 2009 112 Supplement 1 S8 S15 19695211 10.1016/j.actatropica.2009.08.015

[B4] Higgs ES Hayden FG Chotpitayasunondh T Whitworth J Farrar J The Southeast Asian influenza clinical research network: development and challenges for a new multilateral research endeavor Antiviral Res 2008 78 64 68 10.1016/j.antiviral.2007.10.008 18295355

[B5] Whitworth JA Kokwaro G Kinyanjui S Snewin VA Tanner M Walport M Sewankambo N Strengthening capacity for health research in Africa Lancet 2008 372 1590 1593 10.1016/S0140-6736(08)61660-8 18984193 PMC2607030

[B6] The Bamako Call to Action on Research for Health - Strengthening Research for Health, Development, and Equity 2008 Bamako: The Global Ministerial Forum on Research for Health

[B7] Ali R Finlayson A Indox Cancer Research N Ali R Finlayson A Indox Cancer Research N Building capacity for clinical research in developing countries: the INDOX Cancer Research Network experience Glob Health Action 2012 5 10.3402/gha.v5i0.17288 PMC3343878 22566788

[B8] Maher D Biraro S Hosegood V Isingo R Lutalo T Mushati P Ngwira B Nyirenda M Todd J Zaba B Collaborators in ALPHA Network Translating global health research aims into action: the example of the ALPHA network Trop Med Int Health 2010 15 321 328 10.1111/j.1365-3156.2009.02456.x 20070637

[B9] Ramkalawan T Dieppe P Research capacity development and training J Health Serv Res Policy 2008 13 6 11 18806187 10.1258/jhsrp.2008.008008

[B10] Greenwood B Bhasin A Targett G The gates malaria partnership: a consortium approach to malaria research and capacity development Trop Med Int Health 2012 17 558 563 10.1111/j.1365-3156.2012.02970.x 22420422

[B11] Adedokum L Baytop C Goldberg A Gonzales J Wiley A Evaluation of the Fogarty International Center AIDS International Training Research Program (AITRP): Phase II Outcome Evaluation. Final Report 2008 Cambridge, MA: Abt Associates, Inc

[B12] Bennett S Paina L Ssengooba F Waswa D M’Imunya J The impact of Fogarty International Center research training programs on health policy and program development in Kenya and Uganda Submitted for publication to BMC Public Health 10.1186/1471-2458-13-770 PMC3851767 23964653

[B13] Magoha G Speech delivered by the Vice-Chancellor Prof. George Magoha during the 45th graduation ceremony on September 9, 2011 at the Chancellor’s Court 2011 Nairobi: University of Nairobi

[B14] Jentsch B Pilley C Research relationships between the South and the North: Cinderella and the ugly sisters? Soc Sci Med 2003 57 1957 1967 10.1016/S0277-9536(03)00060-1 14499518

[B15] Porter AL Garner J Crowl T Research coordination networks: evidence of the relationship between funded interdisciplinary networking and scholarly impact Bioscience 2012 62 282 288

